# Reducing Caregiver Burden Through Dyadic Support in Palliative Care: A Systematic Review Focused on Middle-Aged and Older Adults

**DOI:** 10.3390/jcm14165804

**Published:** 2025-08-16

**Authors:** Gonçalo Botas, Sara Pires, Cesar Fonseca, Ana Ramos

**Affiliations:** 1 Local Health Unit of Lezírias, 2005-177 Santarém, Portugal; goncalobotasdacruz@campus.esel.pt; 2 Department of Medical-Surgical Nursing, Nursing School of Lisbon (ESEL), 1600-190 Lisbon, Portugal; sarapires@esel.pt; 3 Research, Innovation and Development Centre of Lisbon (CIDNUR), 1600-190 Lisbon, Portugal; 4 Department of Rehabilitation Nursing, University of Évora, 7004-516 Évora, Portugal; cfonseca@uevora.pt; 5 Comprehensive Health Research Centre (CHRC), 7004-516 Évora, Portugal

**Keywords:** caregiver burden, dyadic support, palliative care

## Abstract

**Background/Objectives**: Family caregivers in palliative care often face complex physical, emotional, and logistical challenges, which can result in a significant caregiving burden. Dyadic interventions—designed to support both the patient and the caregiver simultaneously—have emerged as a promising holistic approach to enhancing well-being and quality of life. This systematic review aimed to evaluate the effects of dyadic support interventions in reducing caregiver burden among middle-aged and older adults receiving palliative care. **Methods**: A systematic literature search was conducted following PRISMA guidelines across five databases (CINAHL, MEDLINE, Web of Science, Scopus, and Google Scholar for grey literature) covering the period from 2019 to January 2025. **Results**: Of 653 records identified, 8 studies met the inclusion criteria. Interventions were typically delivered by multidisciplinary teams and included weekly in-person consultations, telephone follow-up, telemedicine, physical exercise sessions, laughter therapy, and music therapy over durations ranging from 16 weeks to 6 months. These programs resulted in reduced anxiety and depressive symptoms (PHQ-4, HADS, SDS, BAI, SAS), improved functional and social performance (SF-36), and/or enhanced quality of life (MQLQ, QOL-AD, KCCQ-12, EORTC QLQ-C30). In patients, they contributed to better symptom control (ESAS, CFS), while in caregivers, they effectively reduced burden (ZBI-12, FCBSI, CBI) and/or supported the anticipatory grief process (PGQ, AGS). However, not all studies reported consistently positive outcomes. **Conclusions**: Structured dyadic interventions that involve both patients and caregivers significantly improve outcomes in palliative care for middle-aged and older adults. Future research should examine their long-term impact and explore the integration of artificial intelligence to optimize intervention delivery.

## 1. Introduction

The global need for palliative care is significantly increasing, driven by an aging population and rising prevalence of chronic diseases. It is estimated that, annually, more than 56.8 million people, including 25.7 million at the end-of-life stage, require palliative care worldwide [1,2,3]. The early integration of palliative care demonstrates benefits for patient and caregiver well-being and for the efficiency of healthcare systems [4,5,6], making it an ethical and fiscally responsible strategy [4].

Frequently, care focuses on the management of physical and emotional symptoms, undervaluing family inclusion and the personalization of care [7,8,9,10]. A systemic approach recognizes that serious illness affects the entire family dynamic [11,12], with communication being a central element for adaptation and emotional balance [13,14,15,16]. The dyad—comprising the patient and primary caregiver—has become a recognized unit of analysis and intervention, particularly within the context of advanced chronic illness [13,17]. This perspective conceptualizes the patient–caregiver relationship as an interconnected unit of care, emphasizing the resultant gains in psychological well-being and shared perceptions of support [13,15]. Informal caregivers, predominantly comprising close family members (spouses, children, siblings) or significant others (friends, neighbors), provide unpaid, non-professional care encompassing the physical, emotional, social, and spiritual dimensions of the palliative care recipient’s life [12,15,18].

Caregiving roles necessitate not only the acquisition of practical skills but also a fundamental reorganization of personal identity and social relationships. This is particularly relevant when caregivers are supporting middle-aged or older adults, who are often presented with frailty, increasing their dependency and morbidity [19]. This transformative process is influenced by a multitude of factors [20], including prior preparation, previous experiences, the availability of support systems, and access to resources, all of which shape the caregiver’s subjective experience of this transition [21]. Transition, in this context, is understood as a dynamic process demanding continuous adjustment, emotional recalibration, and the acquisition of new skills to effectively address emerging challenges [20]. Dyad-focused interventions, particularly within palliative care, have demonstrably positive effects, promoting effective communication, strengthening joint coping mechanisms, and mitigating symptoms of anxiety and depression in informal caregivers [21].

The significant physical, emotional, social, and spiritual demands placed on informal caregivers in palliative care necessitate a multidimensional understanding of caregiver burden [22,23]. This burden encompasses not only the tangible challenges of caregiving but also the psychological distress, unmet needs, and potential vulnerability of social support networks [24], along with the spiritual struggles related to existential issues, hope, and facing mortality [25]. Caregiver burden, therefore, reflects the cumulative impact of these diverse demands, subjectively experienced as negatively affecting the caregiver’s well-being across multiple dimensions [26,27]. Factors such as inadequate preparation, insufficient integration of the caregiver into the therapeutic plan, and a lack of systematic support hinder adaptation, increasing the risk of emotional exhaustion and relational difficulties [28,29]. Furthermore, the degrees of care recipient dependency, caregiver health, family resilience, and social support significantly influence burden levels [29,30,31]. The considerably higher psychological morbidity among end-of-life caregivers compared with the general population (particularly given the extensive time commitment) underscores the critical need for structured, multidimensional interventions [32,33].

While several reviews have examined dyadic interventions within specific populations, such as those with lung cancer [34], or focusing on intervention modalities like dyadic digital psychological interventions [35] and psychosocial interventions [36], a synthesis across diverse populations and intervention types remains lacking. This systematic review aims to identify and synthesize the evidence regarding the effectiveness of dyadic support interventions in reducing caregiver burden among middle-aged and older adults receiving palliative care.

## 2. Materials and Methods

A systematic review was conducted under the Preferred Reporting Items for Systematic Reviews and Meta-Analyses (PRISMA) guidelines to ensure methodological transparency and rigor [37]. Following the recommendations of the Joanna Briggs Institute (JBI) for evidence synthesis [38], the research question was structured using the PICO framework to clearly define the scope of the investigation: which dyadic nursing interventions are effective in reducing caregiver burden among middle-aged and older adults receiving palliative care?

The protocol for this review was prospectively registered with the International Prospective Register of Systematic Reviews (PROSPERO) maintained by the National Institute for Health Research under registration number CRD42024612836 (November 2024).

### 2.1. Search Strategy

Between November 2024 and January 2025, a systematic search was conducted across the following electronic databases: MEDLINE and CINAHL via EBSCOhost and Scopus and Web of Science. A grey literature search was also performed using Google Scholar. A comprehensive and structured search strategy was employed, incorporating descriptors and keywords related to dyadic interventions, caregiver burden, and palliative care. The approach combined controlled vocabulary (MeSH terms) with free-text terms to ensure both sensitivity and specificity. Boolean operators (AND, OR) were used to refine and optimize the search results. The complete search strategy is detailed in Table 1.

Study selection was based on predefined inclusion and exclusion criteria. Duplicate records were automatically removed using the Rayyan© platform. Title and abstract screening were carried out independently by two reviewers (G.B. and A.R.), followed by a full-text review of the selected studies. Disagreements between reviewers were resolved through discussion, and, when necessary, with the involvement of a third reviewer (C.F.). The inter-reviewer agreement rate exceeded 80%, ensuring methodological rigor and consistency throughout the selection process.

### 2.2. Eligibility Criteria

In line with the research objective and methodological framework, specific inclusion and exclusion criteria were established to ensure the rigorous and relevant selection of studies. This review focused on middle-aged and older adults (≥40 years old), in alignment with the target population defined in the research question. Eligible studies were those that addressed caregiver burden within the context of palliative care, specifically examining dyadic interventions involving both the patient and their family caregiver. Only articles published in English, Portuguese, or Spanish between January 2019 and January 2025 were considered, reflecting both the reviewers’ language proficiency and the intention to synthesize the most current evidence available.

Studies with diverse methodological designs were included, particularly quantitative approaches and mixed methods, such as randomized controlled trials (RCTs), quasi-experimental studies, and prospective or retrospective cohort studies, provided they focused on health interventions aimed at reducing caregiver burden through dyadic support. Exclusion criteria involved studies concerning individuals under 40 years of age, opinion papers, editorials, protocols, and secondary literature without original data. Studies that did not focus on caregiver burden, failed to explore dyadic interventions, or were unrelated to the palliative care context were excluded.

### 2.3. Evaluation of Risk of Bias and Methodological Quality of Studies

To ensure methodological rigor and reduce the risk of bias, two independent reviewers (G.B. and A.R.) conducted the full-text screening of all eligible studies. The methodological quality of the included studies was assessed using the Joanna Briggs Institute (JBI) critical appraisal tools, selected according to the specific design of each study. High-quality systematic reviews rely on the rigorous appraisal and synthesis of the best available evidence on the effectiveness of health interventions [39].

Each study was independently evaluated, and scores were calculated based on the percentage of affirmative (“yes”) responses within the appraisal tool. Studies scoring above 80% were classified as high quality, those between 60% and 80% as moderate quality, and those below 60% as low quality [40]. Only studies rated as moderate to high quality were retained for inclusion in this review (see Table 2).

### 2.4. Data Extraction and Synthesis

To ensure systematic and consistent data collection, the authors developed a structured extraction tool to capture essential information from each included study. The data extracted comprised the following variables: authorship, year of publication, country of origin, study objectives, research design, characteristics of the study population, duration and type of intervention, measurement instruments and scales, and main conclusions.

After data extraction, a narrative synthesis was performed to integrate and interpret the relevant evidence emerging from the full-text analysis. A synthesized summary table was constructed to present the health-related outcomes associated with dyadic support interventions aimed at reducing caregiver burden. This table was designed to facilitate a clear and comprehensive understanding of the collective findings.

## 3. Results

### 3.1. Study Selection

**Figure 1:**
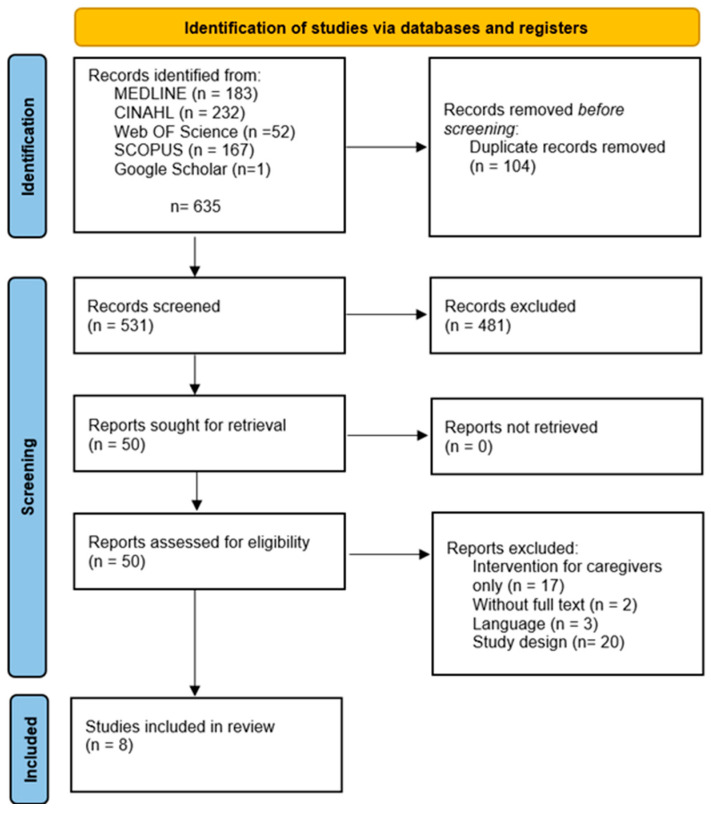
Systematic literature review flowchart.

A total of 635 records were identified through database searches (MEDLINE, CINAHL, Web of Science, Scopus) and one additional record via Google Scholar. After removing 104 duplicates, 531 records were screened by title and abstract, resulting in the exclusion of 481 records. Of those 50 listed, a further 42 were excluded after full-text review for the following reasons: intervention exclusively for caregivers (n = 17), unavailable full text (n = 2), language (n = 3), and unsuitable study design (n = 20). This left a final sample of 8 studies included in the review (Figure 1).

### 3.2. Study Characteristics

Eight studies were analyzed, spanning seven countries (USA, Egypt, Denmark, Taiwan, South Korea, China, and Japan), with the USA represented in two studies. Four study designs were employed: three randomized controlled trials (RCTs), three quasi-experimental studies, one prospective longitudinal study, and one retrospective cohort study. The number of dyads varied significantly across studies, ranging from 6 [46] to 249 [43], with most data collected at baseline and 3, 6, and 12 months, as shown in Table 3.

There was a significant variation in the mean age of patients, ranging from the low 60s to the mid-80s, depending on the study and the specific condition. Similarly, caregiver mean ages varied, but tended to be slightly younger than the patients. The studies analyzed represent a diverse range of healthcare settings and patient populations, reflecting the complexity of providing dyadic care for individuals with multimorbidity. The settings include home-based care [41,42,43], outpatient clinics [44], palliative care wards [45,48], and palliative care outpatient consultations [47]. One study focused on acute hospitalizations [46]. This variety in setting suggests that the need for dyadic support interventions extends across various points in the care continuum and across diverse healthcare environments. The patient populations included individuals with advanced heart failure, various incurable cancers, and Parkinson’s disease-related disorders, highlighting the broad applicability of dyadic support interventions across a range of chronic and life-limiting conditions.

### 3.3. Data Presentation

Table 4 summarizes the key characteristics of the eight studies evaluating dyadic support interventions for patients with serious illness and their caregivers. The studies vary significantly in their interventions (telephone coaching, rehabilitation programs, palliative care consultations, telemedicine support, laughter therapy, music therapy, and a nurse-led care management program), intervention providers (nurses, health professionals, palliative care teams, and specialized therapists), frequency of intervention sessions, assessment instruments used to evaluate outcomes (including measures of caregiver burden, patient quality of life, and emotional distress), and the main results obtained. This overview allows for a comparison of different approaches to dyadic support and their impact on patient and caregiver well-being.

The variety of assessment instruments used in the analyzed studies reflects the complexity of the dimensions being evaluated, both in the patient’s and caregiver’s experiences. The measurement of caregiver burden frequently uses the Zarit Burden Interview (ZBI-12) [41,42,43], demonstrating its relevance and acceptance in the literature. Other studies employed instruments such as the Caregiver Burden Inventory (CBI) [44,48] and the Modified Caregiver Strain Index (MCSI) [42], suggesting different approaches to quantifying this construct. Patient quality of life was assessed using comprehensive questionnaires such as the European Organization for Research and Treatment of Cancer Quality of Life Questionnaire 30 (EORTC QLQ-C30) [44], the EuroQol 5 Dimensions 5 Levels (EQ-5D-5L) [46], and the Kansas City Cardiomyopathy Questionnaire (KCCQ-12) [41], specific to cardiac pathologies. Anxiety and depression were assessed using scales such as the Beck Anxiety Inventory (BAI) [44] and the Hospital Anxiety and Depression Scale (HADS) [42,44], while pain assessment used the Numerical Pain Rating Scale (NPRS) [45]. While the use of multiple instruments allows for a multidimensional assessment, it also presents the challenge of directly comparing results.

Table 5 provides a comparative overview of health-related outcomes in dyadic support interventions for middle-aged and older adults receiving palliative care. The table organizes studies by publication and maps reported outcomes for both patients and caregivers across several domains: anxiety, depressive symptoms, functional and social performance, quality of life, symptom control, burden, and anticipatory grief. A dot indicates that the study measured and reported on that specific outcome. This visual representation allows for a quick identification of each study’s focus, highlights the range of outcomes considered in dyadic support interventions within palliative care, and enables a comparative analysis of measured outcomes across different interventions and study populations.

## 4. Discussion

Collective evidence from recent studies underscores the potential of dyadic and family-centered interventions in mitigating caregiver burden within palliative care settings. A study by Piamjariyakul et al. [41] offers promising evidence on the effectiveness of dyadic interventions, particularly the FamPALcare program, in reducing caregiver burden and improving the well-being of individuals with advanced heart failure. This intervention involved five personalized telephone sessions guided by an educational manual, designed to support family caregivers in managing care at home. Its content emphasized strengthening the bond within the dyad, exploring family beliefs and concerns, engaging caregivers in care planning, and addressing needs in the home context. Specific sessions focused on symptom management in advanced heart failure, advanced directives, legal documentation, and end-of-life communication. A visual representation of the disease trajectory was also provided, though it was perceived as less useful. Overall, the intervention empowered caregivers and contributed to reducing the burden and enhancing the quality of care in the palliative context. In a similar dyad-centered approach, Ibrahim et al. [44] designed a comprehensive palliative care intervention with a rehabilitation component for individuals with incurable cancer and their caregivers. Delivered by a multidisciplinary team, it included physical exercises, psychoeducational sessions, spiritual and existential support, counseling, and caregiver training. For patients, improvements were seen in quality of life, anxiety reduction, and partial relief from depressive symptoms, likely resulting from the combination of emotional sport, tailored physical activity, and spiritual guidance. Among caregivers, positive effects were noted in emotional well-being, functional capacity, and quality of life. A notable reduction in caregiver burden—especially in physical and emotional dimensions—was observed, attributed to structured training, access to validated manuals, and continuous professional support. These two studies [41,44] highlight the effectiveness of multi-component interventions that integrate both educational resources and hands-on, personalized support, suggesting that a combination of practical training and emotional coaching is a key driver of positive outcomes.

The psychological intervention evaluated by von Heymann et al. [43] in the design domain was integrated into home-based palliative care and targeted dyads coping with incurable cancer. The program included early sessions to assess dyadic needs and build therapeutic rapport, followed by flexible, individualized, or dyadic sessions tailored to specific challenges. While maintaining oncological and nursing care, this intervention aimed to provide psychosocial support. However, it did not result in statistically significant reductions in caregiver burden, as measured by ZBI-12 over 6 months. The small effect sizes suggest limited impact, potentially due to the multifaceted nature of caregiver burden, which encompasses emotional, physical, social, and existential stressors. The intervention’s structure may have lacked sufficient intensity, frequency, or timing to produce measurable improvements, especially in caregivers already experiencing high emotional fatigue. It also underscores a critical challenge in dyadic care: measuring effectiveness, as ZBI-12 may not have been sensitive enough to capture subtle but meaningful changes in burden. In contrast, Wu et al. [47] assessed a multidisciplinary Palliative Care Consultation Service (PCCS) for dyads facing progressive, incurable illnesses. Delivered through initial assessments and weekly follow-ups, this intervention was tailored to individual needs and supported by regular interdisciplinary reviews. It aimed to address symptom control and care-related concerns across oncological and non-oncological populations. Over 14 days, caregiver burden was progressively reduced, as shown by the Family Caregiver Burnout Scale (FCBS). Notably, caregivers of cancer patients experienced relief across physical, psychological, and spiritual domains, while those caring for non-cancer patients saw improvements in daily demands and economic stress. A secondary finding suggested that religious caregivers experienced less physical and psychological burden, hinting at the potential buffering role of spiritual coping.

Kluger et al. [42] explored the integration of palliative care in neurology through a telemedicine-based intervention for individuals with Parkinson’s disease and related disorders. The program aimed to improve patient quality of life while reducing caregiver burden by training neurologists in palliative principles and offering continuous remote dyadic support. However, the results revealed an increase in caregiver burden in the intervention group for 12 months, as measured by ZBI-12 and MCSI, compared with usual care. Despite a slight improvement in 6 months (CGIC), this was not sustained. The increase in burden may reflect heightened caregiver awareness of the complexity of care, without parallel strengthening of coping mechanisms. It highlights the challenges of caregiving in neurodegenerative conditions, where support needs are prolonged and multifaceted. The contrasting outcomes of this telemedicine-based model compared with others like FamPALcare [41] (telephone coaching) and PCCS [47] (in-person multidisciplinary support) suggest that the mode of delivery alone is not sufficient. The Kluger et al. [42] study reveals a critical paradox: increasing awareness of care needs without providing effective tools to manage them can exacerbate caregiver strain, a finding that should inform the design of future interventions. A study by Moon et al. [45] introduced a psychosocial intervention—laughter therapy—for family caregivers of individuals with advanced cancer in palliative care. Conducted in a hospital setting through brief, daily sessions led by trained professionals, the intervention significantly reduced emotional and psychological caregiver burden. While the control group reported worsening outcomes, participants in the laughter therapy group experienced improved emotional well-being and relief from care-related demands, demonstrating the potential of simple, cost-effective emotional support strategies in high-stress care environments. Similarly, Ma and Bai [48] investigated family music therapy as a non-pharmacological intervention. Daily music listening sessions—customized to individual preferences and delivered via WeChat—were accompanied by guidance on posture and volume and supported by a multidisciplinary team including music therapists. The intervention led to significant improvements in sleep quality, reduced anticipatory grief, and a marked decrease in caregiver burden. These findings support the feasibility and efficacy of music-based interventions in easing the emotional toll of caregiving in palliative settings.

Lastly, the BOLSTER intervention developed by Pozzar et al. [46] focused on supporting patients with gynecologic cancer-associated peritoneal carcinomatosis and their caregivers’ post-hospitalization. Led by nurses, it combined structured telephone support with a mobile app for symptom monitoring. Educational components addressed symptom relief, medical device use, and self-care preparation. Caregivers reported enhanced confidence in care tasks, improved communication with healthcare teams, and better support for decision making. Despite the complex and emotionally demanding context, BOLSTER emerged as a promising strategy to reduce both emotional and practical burdens.

Collectively, these studies highlight the importance of personalized, multi-component interventions that target the patient–caregiver dyad. Interventions that integrate education, emotional support, and practical skill building, especially when delivered continuously and flexibly, appear most effective in mitigating caregiver burden. A large portion of the positive findings stemmed from studies with observational designs, such as those by Ibrahim et al. [44], Ma et al. [48], and Pozzar et al. [46]. While these results are promising, it is important to note that such studies are more susceptible to bias and confounding factors, which may have inflated the magnitude of the observed effects. The methodological and contextual heterogeneity among the studies—ranging from laughter and music therapies to telemedicine and comprehensive rehabilitation programs—makes it difficult to isolate which specific components are most effective. Findings from the more rigorous studies, specifically the randomized controlled trials (RCTs), suggest that the effectiveness of dyadic interventions is not unequivocally demonstrated. Success appears to be contingent upon a careful combination of factors, such as intensity, duration, and type of support, which must be meticulously aligned with the specific needs of the patient and caregiver.

## 5. Limitations

Despite the valuable insights offered by this systematic review, some limitations must be acknowledged. First, there was considerable heterogeneity among the included studies in terms of intervention types, duration, intensity, and outcome measures, which limits the comparability of findings and impedes the possibility of conducting a meta-analysis. Interventions ranged from structured educational sessions to psychosocial and spiritual support strategies, with varying degrees of professional involvement and delivery formats (e.g., face-to-face, telehealth, app-based). This diversity, while reflective of real-world practices, poses challenges in drawing generalized conclusions. Second, while some studies reported significant reductions in caregiver burden, others showed limited or no measurable effects. In some cases, small sample sizes, short follow-up periods, and potential ceiling effects may have reduced the ability to detect meaningful changes, particularly in highly burdened caregivers where the strain is persistent and multifactorial.

Most studies focused on cancer populations, potentially limiting the applicability of findings to caregivers of individuals with other chronic diseases, whose trajectories and support needs differ substantially. The cultural and contextual differences across studies, including variations in healthcare systems, family dynamics, and religious or spiritual coping, may influence both the design and effectiveness of dyadic interventions. This diversity highlights the need for culturally sensitive and context-specific approaches in future research.

## 6. Conclusions

This systematic review demonstrated that structured dyadic interventions—simultaneously involving the patient receiving palliative care and their informal caregiver—constitute a promising strategy to alleviate the emotional, physical, and practical burdens of caregiving, particularly in the context of advanced chronic illness in middle-aged and older adults. The studies consistently revealed significant improvements in patients’ quality of life, symptom control, and psychosocial well-being, as well as a meaningful reduction in caregiver burden and anticipatory grief. The interventions analyzed included weekly in-person sessions, telephone follow-ups, telemedicine programs, adapted physical activity, psychological support (including laughter and music therapy), structured training, and digital resources. These multidimensional approaches, generally implemented by interdisciplinary teams, proved effective when delivered continuously, flexibly, and in a manner centered on the dyad’s needs. Moreover, consistent themes suggest that strengthening communication, actively involving caregivers in care planning, and providing emotional and educational support are important components of these successful interventions.

Therefore, future research is encouraged to explore the long-term effectiveness of these interventions and their adaptability to different clinical, cultural, and socioeconomic contexts. While not directly evaluated in this review, the integration of emerging digital technologies, particularly artificial intelligence-based solutions, could contribute to more personalized, predictive, and scalable monitoring, thereby optimizing the delivery and impact of these support strategies.

## Figures and Tables

**Table 1:** Search strategy conducted in MEDLINE Complete (EBSCOhost), CINAHL Complete (EBSCOhost), Scopus, and Web of Science.

Search	Descriptors
#1	“Dyad*” OR “Caregivers” OR “Family Caregivers” OR “Informal Caregivers” OR “Spouse Caregivers” OR “Carers”
#2	“Palliative care” OR “Hospice and Palliative Care Nursing”
#3	“Caregiver Burden” OR “Caregiver Burnout” OR “Caregiver Stress” OR “Caregiving Stress” OR “Sickness Impact Profile” OR “Symptom Burden”
#4	[(“Dyad*” OR “Caregivers” OR “Family Caregivers” OR “Informal Caregivers” OR “Spouse Caregivers” OR “Carers”) AND (“Palliative care” OR “Hospice and Palliative Care Nursing”) AND (“Caregiver Burden” OR “Caregiver Burnout” OR “Caregiver Stress” OR “Caregiving Stress” OR “Sickness Impact Profile” OR “Symptom Burden”)]

**Table 2:** Results of the methodological quality assessment of the included manuscripts.

**JBI Critical Appraisal Tool for Randomized Controlled Trials**		
**Study**	**Score**	**Level**
Piamjariyakul et al., 2024 [41]	84.6%	High
Kluger et al., 2023 [42]	84.6%	High
von Heymann et al., 2023 [43]	76.9%	Moderate
**JBI Critical Appraisal Tool of Quasi-Experimental Studies**		
**Study**	**Score**	**Level**
Ibrahim et al., 2024 [44]	77.8%	Moderate
Moon et al., 2022 [45]	77.8%	Moderate
Pozzar et al., 2022 [46]	77.8%	Moderate
**JBI Critical Appraisal Tool of Analytical Cross-Sectional Studies**		
**Study**	**Score**	**Level**
Wu et al., 2020 [47]	100.0%	High
**JBI Critical Appraisal Tool of Analytical Cohort Studies**		
**Study**	**Score**	**Level**
Ma et al., 2024 [48]	87.5%	High

**Table 3:** The main characteristic of the included studies.

Publication	Country	Study Aim	Study Design	Setting	Sample/Participants	Data Assessment
Piamjariyakul et al., 2024 [41]	USA	To evaluate the effectiveness of the FamPALcare intervention program, which involves caring for individuals diagnosed with advanced heart failure and their family caregivers, including outcomes related to caregiver burden	Randomized controlled trial	At home, in rural communities in the Appalachian region	A total of 39 dyads. In the intervention group, 21 dyads; in the control group, 18 dyads. A total of 11 dyads did not complete the program (28%). Patients had a mean age of 65.66 years, with 66.7% male and 33.3% female. Family caregivers had a mean age of 62.05 years.	Baseline, 3, and 6 months
Ibrahim et al., 2024 [44]	Egypt	To assess the impact of a comprehensive palliative rehabilitation care program on the quality of life of individuals with incurable cancer and their family caregivers	Quasi-experimental study	Outpatient clinic of an oncology center	A total of 88 dyads. Patients had a mean age of 65.79 years, with 54.5% male. Family caregivers had a mean age of 42.05 years, with 49.1% female.	Pre- and post-intervention
von Heymann et al., 2023 [43]	Denmark	To evaluate the effect of specialized home-based palliative care, enhanced with a dyadic psychological intervention, on reducing the burden among family caregivers of patients with incurable cancer	Randomized controlled trial		A total of 249 dyads: 134 in the intervention group, 115 in the control group. Family caregivers: Intervention group—mean age 61 years, 63% female, 77% spouse/partner. Control group—mean age 62 years, 65% female, 80% spouse/partner.	2, 4, and 6 weeks and at 6 months
Wu et al., 2020 [47]	Taiwan	To assess the effectiveness of the Palliative Care Consultation Service (PCCS) in reducing the burden on family caregivers of patients with incurable progressive diseases, comparing oncological and non-oncological conditions	Prospective longitudinal study	Palliative care outpatient consultation at a medical center	A total of 68 dyads: 46 oncological; 22 non-oncological. Demographics (oncological): Patients—mean age 67.6 years (SD 15.3), 56.5% male. Caregivers—mean age 52.6 years (SD 10.5), 73.9% female, 65.2% not spouse. Demographics (non-oncological): Patients—mean age 83.6 years (SD 11), 50% male. Caregivers—mean age 56.4 years (SD 11.8), 79.1% female, 81.8% not spouse.	Baseline, day 7, and day 14.
Kluger et al., 2023 [42]	USA	To evaluate whether palliative care education for community neurologists, combined with telemedicine support, improves the quality of life of persons with Parkinson’s disease and related disorders (PDRDs) and reduces caregiver burden	Randomized controlled trial	Participants were discharged from hospitals in California, Colorado, and Wyoming	A total of 359 persons with PDRDs (179 intervention, 180 control), 300 family caregivers (143 intervention, 157 control), 143 dyads per group. Demographics (patients): Intervention—mean age 73.6 (SD 9.1), 62% male. Control—mean age 74.4 (SD 7.6), 67.8% male. Demographics (caregivers): Intervention—mean age 65.8 (SD 12.1), 78.2% female, 72% spouse/partner, 21% child, 7% other. Control—mean age 69.2 (SD 10.3), 65% female, 82.1% spouse/partner, 11.5% child, 6.4% other.	6 and 12 moths
Moon et al., 2022 [45]	South Korea	To evaluate the effects of laughter therapy on mood disturbance and pain in patients with terminal cancer and caregiver burden in family caregivers	Quasi-experimental study	Palliative care ward in a tertiary university hospital	A total of 49 dyads (26 in intervention, 23 in control). Patients (intervention): mean age 61.04 (SD = 12.61), 76.9% male. Patients (control): mean age 60.83 (SD = 10.63), 69.6% male. Caregivers (intervention): mean age 50.19 (SD = 16.51), 80.8% female. Caregivers (control): mean age 55.35 (SD = 12.59), 56.5% female.	Pre- and post-intervention
Ma et al., 2024 [48]	China	To evaluate the effectiveness of family music therapy in reducing emotional and physical distress in advanced hepatocellular carcinoma patients in palliative care and their family caregivers	Retrospective cohort study	Palliative care ward	A total of 120 dyads (65 intervention, 55 control). Patients aged 18–80 with stage III–IV hepatocellular carcinoma. Intervention group (mean age 62.6 years, 64.6% male), control group (mean age 66.7 years, 54.5% male). Caregivers: intervention group (mean age 46 years, 73.9% female), control group (mean age 48 years, 74.5% female).	Pre- and post-intervention
Pozzar et al., 2022 [46]	USA	To develop and field-test a nurse-led care management intervention (BOLSTER) to support patients and caregivers following hospitalization for gynecologic cancer-associated peritoneal carcinomatosis	Quasi-experimental study	Acute hospitalizations	A total of 6 dyads: 6 patients were an average of 64 (SD = 7.31) years old, and 6 caregivers were an average of 64 (SD = 6.63) years old.	Pre- and post-intervention

**Table 4:** Description of the dyadic support programs to reduce burden in palliative care.

Publication	Intervention Providers	Dyadic Support Program Content	Intervention Frequency	Instruments	Main Results
Piamjariyakul et al., 2024 [41]	Nurses	Named FamPALcare, the intervention includes telephone coaching focused on education, emotional support, and advanced care planning. The intervention group received a manual to follow during sessions, an assessment of family beliefs and concerns, guidance on caregiver involvement, and identification of home care needs.	A total of 5 telephone sessions lasting 60 to 90 min	Zarit Burden Interview (ZBI-12), Patient Health Questionnaire (PHQ-4), Kansas City Cardiomyopathy Questionnaire (KCCQ-12), Helpfulness Rating Scale (HRS-11)	The most valued aspects were strategies for managing advanced heart failure symptoms, information about advanced directives and legal documents, and the comfort of discussing care options with family and healthcare professionals. The disease trajectory chart was considered less useful. Caregivers showed a progressive reduction in burden over 6 months, along with decreased symptoms of anxiety and depression. Patients demonstrated improvement in health status and quality of life, with consistent progress from the 3-month mark.
Ibrahim et al., 2024 [44]	Health professionals qualified in physiotherapy, psychoeducation, counselling, and spiritual care	A rehabilitation program that encompassed physical exercise, psychological education, one-on-one support sessions, and, notably, elements focused on spiritual and existential matters.	A total of 16 sessions, between 10 and 60 min	Beck Anxiety Inventory (BAI), Caregiver Burden Inventory (CBI), European Organization for the Research and Treatment of Cancer Quality of Life Questionnaire 30 (EORTC QLQ-C30), Hospital Anxiety and Depression Scale (HADS), Short Form Health Survey (SF-36)	A sharp reduction in anxiety levels and a more moderate decrease in depressive symptoms were observed. Significant improvements were noted across various domains of quality of life, including physical capacity, functional performance, emotional role, and mental health, with slight progress in social functioning. There was also an overall reduction in caregiver burden, particularly with improvements in the physical and emotional aspects of caregiving.
von Heymann et al., 2023 [43]	Specialized palliative care nurses	The initial two sessions focused on needs assessment and therapeutic alliance. Follow-up sessions were dyadic or individual, tailored to each dyad’s needs. Oncology treatments and home care continued concurrently.	First 2 sessions within the first month; subsequent sessions scheduled Flexibly over 6 months according to dyad needs	Zarit Burden Interview (ZBI-12)	No significant effect on overall caregiver burden. Small effect sizes were observed (from 0.35 to −0.85) on total burden, personal strain, and role strain subscales.
Wu et al., 2020 [47]	Palliative care team (specialists, palliative nurses, social workers, chaplain)	Palliative Care Consultation Service (PCCS)—initial assessment by physician and nurse, follow-up, multidisciplinary weekly meetings and care planning.	Follow-up 1–2x/week; service ends upon symptom control, resolution of problems, or transfer/discharge/death	Symptom Distress Scale—patients (SDS-CMF), Family Caregiver Burden Scale (FCBS)	Caregiver burden (FCBS) decreased in both groups. Greater reduction in physical–psychological and spiritual burden for oncological caregivers; greater reduction in daily activity and financial burden for non-oncological caregivers.
Kluger et al., 2023 [42]	Community neurologists and a specialized palliative care team via telemedicine	Telemedicine-based palliative care. Included education for neurologists and additional support to the dyad (person and caregiver) via a telemedicine team. Follow-up and decision-making support provided remotely.	12 months	Zarit Burden Interview (ZBI-12), Quality of Life in Alzheimer’s Disease (QOL-AD—patient), Edmonton Symptom Assessment Scale (ESAS—patient), Hospital Anxiety and Depression Scale (HADS—patient and caregiver), McGill Quality of Life Questionnaire (MQLQ—patient), Prolonged Grief Questionnaire (PGQ), Modified Caregiver Strain Index (MCSI), Functional Assessment of Chronic Illness Therapy—Spiritual Well-being Scale (FACIT—caregiver)	While the intervention improved the perceived clinical trajectory at 6 months, it did not reduce caregiver burden. Some indicators of burden and strain increased over time in the intervention group. This may reflect a greater awareness of caregiver challenges resulting from closer engagement with palliative care teams, rather than a failure of the intervention itself.
Moon et al., 2022 [45]	A total of 2 certified laughter therapy specialists and 1 nursing professor (oncology care)	The laughter therapy program was conducted with groups of 3 to 4 pairs at a time, either in the morning or in the afternoon, in a private room within the hospice ward.	Daily sessions of 20–30 min for 5 consecutive days	Linear Analogue Self-Assessment (LASA—patients and caregivers, Numerical Pain Rating Scale (NPRS), Burden Measure (BM)	The intervention group showed a significant reduction in caregiver burden, emotional distress in both patients and caregivers, and pain levels in patients. In contrast, the control group experienced increases in all these outcomes.
Ma et al., 2024 [48]	Palliative care team and music therapists	Family music therapy via WeChat: 30 min daily sessions of relaxing and uplifting music for 4 weeks, with guidance on listening posture and volume.	A total of 30 min each time once a day, and the treatment lasting for 4 weeks	Self-Rating Depression Scale (SDS), Self-Rating Anxiety Scale (SAS), Cancer-Related Fatigue Scale (CFS), Pittsburgh Sleep Quality Index (PSQI), Anticipatory Grief Scale (AGS), Caregiver Burden Inventory (CBI)	Patients showed significant reductions in depression, anxiety, and fatigue. Caregivers had improved sleep quality, reduced anticipatory grief, and decreased caregiver burden.
Pozzar et al., 2022 [46]	Nursing and oncologists	BOLSTER program with nursing visits, starting at home and continuing via telehealth or phone or in person. Symptom monitoring, personalized education, goal setting, digital support through a mobile app, printed materials, and educational videos.	Intervention of 10 weeks comprising 12 nurse visits	EuroQol 5 Dimensions 5 Levels (EQ-5D-5L), Functional Assessment of Chronic Illness Therapy—Palliative Care (FACIT-PAL), Hospital Anxiety and Depression Scale (HADS), Caregiver Reaction Assessment (CRA)	All patients and caregivers who completed interviews recommended the BOLSTER program, expressed satisfaction with the sessions, and felt it helped patients understand their illness. Most also agreed it improved symptoms, supported coping, and assisted with future planning.

**Table 5:** Health-related outcomes of dyadic support in palliative care focused on middle-aged and older adults.

Publication	Patient and Caregiver				Patient	Caregiver	
	Anxiety	Depressive Symptoms	Functional and Social Performance	Quality of Life	Symptom Control	Burden	Anticipatory Grief
Piamjariyakul et al., 2024 [41]	(+)	(+)		(+)	(+)	(+)	
Ibrahim et al., 2024 [44]	(+)		(+)	(+)		(+)	
von Heymann et al., 2023 [43]						(0)	
Wu et al., 2020 [47]		(+)				(+)	
Moon et al., 2022 [45]		(+)			(+)	(+)	
Ma et al., 2024 [48]	(+)				(+)	(+)	
Pozzar et al., 2022 [46]			(+)		(+)	(+)	(+)
Kluger, et al., 2023 [42]						(-)	

*Legend:* (+) positive effect; (0) no significant effect; (-) negative effect.

## Data Availability

No new data were created or analyzed in this study.
